# A Novel Scheme for High-Accuracy Frequency Estimation in Non-Contact Heart Rate Detection Based on Multi-Dimensional Accumulation and FIIB

**DOI:** 10.3390/s25165097

**Published:** 2025-08-16

**Authors:** Shiqing Tang, Yunxue Liu, Jinwei Wang, Shie Wu, Xuefei Dong, Min Zhou

**Affiliations:** 1 School of Physics and Electronic Information, Yantai University, Yantai 264000, China; tangshiqing@s.ytu.edu.cn (S.T.); wushie@ytu.edu.cn (S.W.); dongxuefei@s.ytu.edu.cn (X.D.); 2 College of Electronic and Information Engineering, Nanjing University of Aeronautics and Astronautics, Nanjing 210016, China; zhoumin_zym@nuaa.edu.cn

**Keywords:** FIIB, accumulation, frequency spectrum analysis, FMCW, non-contact heart rate detection

## Abstract

This paper proposes a novel heart rate detection scheme to address key challenges in millimeter-wave radar-based vital sign monitoring, including weak signals, various types of interference, and the demand for high-precision and super-resolution frequency estimation under practical computational constraints. First, we propose a multi-dimensional coherent accumulation (MDCA) method to enhance the signal-to-noise ratio (SNR) by fully utilizing both spatial information from multiple receiving channels and temporal information from adjacent range bins. Additionally, we are the first to apply the fast iterative interpolated beamforming (FIIB) algorithm to radar-based heart rate detection, enabling super-resolution frequency estimation with low computational complexity. Compared to the traditional fast Fourier transform (FFT) method, the FIIB achieves an improvement of 1.08 beats per minute (bpm). A reordering strategy is also introduced to mitigate potential misjudgments by FIIB. Key parameters of FIIB, including the number of frequency components *L* and the number of iterations *Q*, are analyzed and recommended. Dozens of subjects were recruited for experiments, and the root mean square error (RMSE) of heart rate estimation was less than 1.12 bpm on average at a distance of 1 m. Extensive experiments validate the high accuracy and robust performance of the proposed framework in heart rate estimation.

## 1. Introduction

Heart rate (HR) monitoring is essential for assessing cardiac health and overall well-being, particularly in individuals with chronic conditions such as hypertension, diabetes, and chronic obstructive pulmonary disease (COPD) [1,2]. Moreover, abnormal fluctuations in HR can indicate cardiovascular risks or mental health conditions such as depression [3,4]. Current HR monitoring methods mainly include manual, wearable, and non-contact approaches [5]. Although traditional techniques such as pulse counting and photoplethysmography (PPG)-based devices [6,7] are widely used, they may cause discomfort or be unsuitable for sensitive individuals, infants, or the elderly. In contrast, non-contact monitoring provides a more comfortable and non-invasive solution, enabling continuous and remote tracking in both clinical and home environments [8].

Radar systems provide a promising approach for detecting environmental dynamics. By transmitting electromagnetic waves and analyzing the reflected signals, radar can detect subtle movements. Continuous wave (CW) radar, characterized by its simple architecture and low power consumption, has been widely used for vital sign monitoring [9,10,11]. However, CW radar cannot measure distance and suffers from a limited dynamic range [12]. To address these limitations, advanced radar technologies such as ultra-wideband (UWB) and frequency-modulated continuous wave (FMCW) have been developed. UWB provides high range accuracy but is limited by power and sampling constraints. In contrast, FMCW radar combines the sensitivity of CW with superior range resolution, multi-target discrimination, and strong anti-interference capabilities. Recently, millimeter-wave FMCW radar has attracted increasing attention for short-range vital sign detection [13,14,15,16].

Radar-based vital sign signal processing primarily relies on the phase analysis of reflected signals. However, heartbeat signals are extremely weak and easily masked by noise, making it necessary to enhance the SNR and detection accuracy. In the signal processing pipeline, spatial domain accumulation plays a crucial role as it effectively utilizes information from multiple antennas to improve the SNR. For example, [17] proposed a minimum distortion method that employs multi-channel fusion to enhance the SNR of received signals using spatial information; however, it is only applicable to UWB radar and not suitable for FMCW radar. Christoph Bruser et al. [18] proposed two algorithms for estimating HR from multichannel vibration signals, demonstrating that both outperformed their single-channel counterparts. However, these methods were developed for use with multiple force sensors rather than for spatial domain signal processing. In [19], Zekun Chen et al. enhanced the SNR of phase signals using a multi-channel averaging (MCA) technique based on FMCW radar. However, vital signals from different receiving channels often lack consistency, leading to low inter-channel correlation. Moreover, since phase unwrapping is a nonlinear operation, simply summing multi-channel signals does not always yield desirable results. Few existing studies have explored the use of multi-channel accumulation methods. Inspired by the principle of “fewer but better,” we propose a method that uses correlation coefficients to select a subset of high-quality receiving channels for accumulation, thereby significantly improving the SNR of vital sign signals.

In terms of temporal domain accumulation, G. Beltrão et al. proposed a novel slow-time phase correlation processing method, which effectively accumulated vital sign energy around selected range bins [20]. Literature [21,22] explored methods for signal accumulation using multiple range bins or multiple regions, respectively. Z. Wang et al. introduced an energy aggregation analysis (EAA) method for sector and distance selection in human localization, along with a multicell correlation fusion (MCF) method, enhancing the accuracy of respiration and HR detection [23]. Reference [24] proposed a phase accumulation linear interpolation (PA-LI) method using millimeter-wave FMCW radar, which effectively reduced phase noise and improved the SNR of vital sign signals. T. Pan et al. [25] suggested a time-domain coherent accumulation (TDCA)-based variational mode decomposition (VMD) method for detecting vital sign signals. Literature [26] fused the chirp information within each frame and cascaded multiple data frames into a long-frame matrix for the purpose of localizing the human body using energy features. The above schemes can enhance the signal energy and improve the SNR by utilizing signal accumulation methods in either the temporal or spatial domain. However, there is not a scheme based on joint accumulation in the temporal and spatial domains. Obviously, signal accumulation in a single domain does not fully utilize the information potential from multi-antennas and multiple range bins, so it is difficult to achieve the optimal improvement of SNR.

Several existing studies have applied relatively simple denoising methods such as digital filtering and wavelet transform to millimeter-wave radar-based vital sign detection. For example, Liu et al. introduced the use of cascaded bandpass filters to separate respiration and heartbeat signals [27], offering computational simplicity but limited effectiveness in scenarios with overlapping frequency components. Another work by Dai et al. utilized wavelet transform for multi-scale analysis of non-stationary vital sign signals [28], providing better adaptability but relying heavily on the choice of mother wavelet and decomposition level. Compared with these methods, VMD adaptively decomposes signals into intrinsic mode functions without requiring predefined basis functions, enabling effective separation of overlapping respiratory and cardiac components. Although VMD introduces additional computational complexity, it has been widely adopted in recent heart rate detection studies due to its robustness and superior performance in low SNR environments [7,29,30]. Therefore, considering the trade-offs between computational cost and denoising effectiveness, we selected VMD as the core method in this study to achieve more accurate heartbeat detection.

Moreover, the fundamental component of the heart rate signal is often contaminated by respiratory harmonics, cross-modulation, and other interference. In the frequency domain, these interference components are often located close to the HR fundamental frequency, making effective separation difficult for conventional spectral analysis methods and ultimately degrading HR detection accuracy. Therefore, a frequency estimation method with high resolution, high accuracy, and low computational complexity is essential. FFT-based frequency estimation is the most basic and widely used approach [13,14,31]. However, it has significant limitations in frequency accuracy and lacks super-resolution capability. To address these issues, the zero-attracting sign exponentially forgetting least mean square (ZA-SEFLMS) algorithm was proposed in [32] to achieve a high-resolution sparse spectrum for accurate RR and HR estimation. However, this method incurs high computational cost, and its convergence speed may be reduced when handling complex signals. C.-H. Hsieh et al. proposed the harmonic MUSIC (HMUSIC) algorithm [33], which utilizes harmonic components for frequency estimation of vital signals. Although HMUSIC inherits the super-resolution capability of the original Multiple Signal Classification (MUSIC) algorithm, it also suffers from high computational requirements, making it difficult to implement in practical engineering applications. To overcome these limitations, we adopt the FIIB algorithm. Compared to FFT and MUSIC, FIIB combines super-resolution capability, high estimation accuracy, and low computational complexity, making it particularly suitable for practical HR detection.

In this paper, we propose a novel vital sign detection framework that integrates MDCA with the FIIB algorithm to achieve accurate heart rate estimation. The key contributions of this work are summarized as follows:

1. MDCA for SNR enhancement: We propose a two-dimensional accumulation method. In the spatial domain, a channel with the largest energy is selected as the reference channel, and then an optimal channel that has the highest correlation with the reference channel is selected. Then, vital sign signals from these two channels are added for accumulation. Subsequently, temporal domain accumulation is performed. A range bin with the largest energy and the one with the largest energy among the adjacent range bins are selected for accumulation. Finally, the temporal–spatial domain accumulated signals are summed to complete the joint temporal–spatial domain accumulation.
2. FIIB for high-resolution frequency estimation: Following heartbeat signal extraction using the VMD method [34], we apply the FIIB algorithm for spectral analysis. Owing to its super-resolution capability and high estimation accuracy, FIIB significantly improves HR measurement performance without imposing a substantial computational burden.
3. FIIB amendment and parameter selection: Since FIIB is newly introduced to radar-based vital sign detection, we propose an amplitude-based reordering strategy to mitigate potential spectral misjudgments. In addition, we analyze key algorithm parameters, including the number of frequency components *L* and iterations *Q*, and provide experimentally validated guidelines for their selection.

The remainder of this paper is organized as follows: Section 2 introduces the working principles of FMCW radar and presents the system model. Section 3 describes the proposed signal processing framework, including the MDCA and FIIB algorithms. Section 4 provides experimental validation and performance analysis. Finally, Section 5 concludes the paper.

## 2. System Model

**Figure 1:**
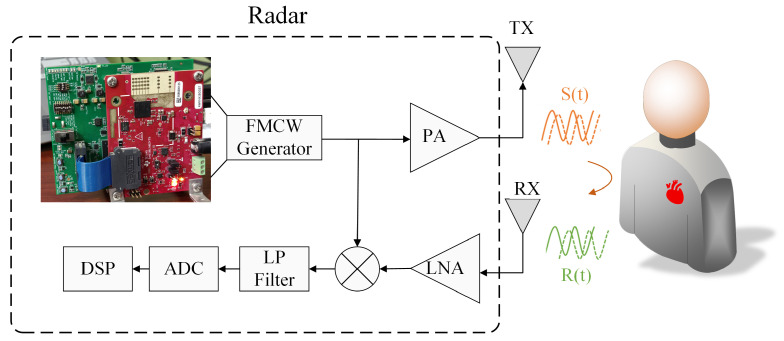
Block diagram of the FMCW radar system.

The radar signals pass through an analog-to-digital converter (ADC) and are subsequently sent to a digital signal processor (DSP) for further processing. The basic working principle is illustrated in Figure 1.

In an FMCW radar system, the transmitted signal is a linearly frequency-modulated (FM) chirp:

(1) $$S\left(t\right)=Ae^{j2\pi (f_{c}t+\frac{B}{2T}t^{2})}e^{j\varphi_{0}\left(t\right)}$$

where *A* denotes the signal amplitude, $f_{c}$ is the operating frequency (i.e., initial frequency of the chirp), *B* is the pulse bandwidth, *T* is the pulse duration, and $\varphi_{0}\left(t\right)$ represents the initial phase. When the transmitted signal encounters a human body or other objects, it generates a reflected signal R(t), which can be expressed as:

(2) R(t)=αS(t−τ)

where α denotes the attenuation coefficient, τ=2d/c is the time delay of the object at a radial distance *d*, and *c* represents the speed of light. The radar mixes the received signal R(t) with the transmitted signal S(t) to obtain the intermediate frequency (IF) signal $S_{IF}\left(t\right)$, which is defined by the following equation:

(3) $$S_{IF}\left(t\right)=S\left(t\right)R^{*}\left(t\right)=\alpha A^{2}e^{j2\pi (f_{c}+\frac{B}{2T}t)\tau }e^{j\phi (t-\tau )}$$

where $R^{*}\left(t\right)$ is the complex conjugate form of R(t). Note that $\tau^{2}$ is ignored in the calculations because it is too small. For short-range applications, $\varphi_{0}\left(t\right)$ is negligible. ϕ(t) represents the time-varying phase of the monitored target, and its expression can be written as:

(4) $$\phi \left(t\right)=\frac{4\pi d(t)}{\lambda_{c}}$$

where $\lambda_{c}=c/f_{c}$ is the radar operating wavelength. As the target moves slightly within the radar beam, the phase changes accordingly, reflecting these micro-motions.

For a target object located at a nominal range $d_{0}$, the equivalent phase can be expressed as:

(5) $$\phi \left(t\right)=\frac{4\pi d_{0}}{\lambda_{c}}+\frac{4\pi x(t)}{\lambda_{c}}$$

where $x\left(t\right)=x_{r}\left(t\right)+x_{h}\left(t\right)$ represents displacements caused by respiration $x_{r}\left(t\right)$ and heartbeat $x_{h}\left(t\right)$. These displacements contain patterns of motion from multiple sources, not only from the surface of the chest wall, but also from the abdomen, shoulders, back, and other parts of the body [35]. By accurately recovering chest wall displacements x(t) and analyzing the periodicity of the signals, it is possible to precisely estimate respiration and heart rate [36].

## 3. Vital Signals Processing

**Figure 2:**
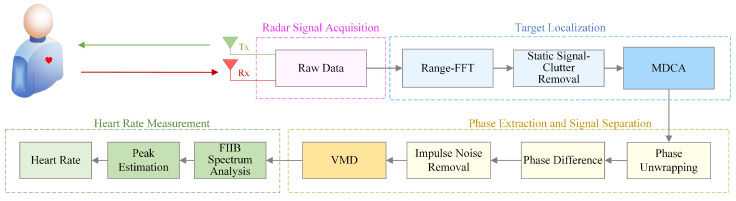
Block diagram of HR monitoring based on FMCW radar.

In this study, we propose a novel scheme for accurate HR detection using 77 GHz FMCW radar. Figure 2 illustrates the block diagram of the proposed system, which comprises three main components: signal preprocessing, signal decomposition, and HR estimation.

During signal preprocessing, the phase signal corresponding to the target range bin is extracted through Range-FFT and static clutter removal. The proposed MDCA method is then applied to adaptively select the optimal receive channel and range bin. Subsequent steps include phase unwrapping, phase difference computation, and impulse noise suppression to extract the vital sign signal.

Next, the VMD algorithm is applied to the preprocessed signal to separate the heartbeat component, completing the signal decomposition stage. Finally, the FIIB algorithm is employed in this context to estimate the heart rate. Notably, FIIB performs both coarse and fine frequency estimations, significantly improving measurement precision and frequency resolution.

This processing framework effectively enhances the SNR and mitigates various interferences in complex indoor environments through a multi-stage procedure, thereby achieving high-accuracy HR estimation. The following subsections detail each processing step.

### 3.1. Multi-Dimensional Coherent Accumulation Algorithm

Radar-detected heartbeat signals are extremely weak and easily affected by respiratory harmonics, cross-modulation, and noise. Signal accumulation is thus essential for enhancing signal quality and enabling accurate HR estimation. Although many modern radars (e.g., TI, NXP, and other manufacturers) support multi-channel reception, most methods of HR detection still use single-channel processing, underutilizing available information. In the following, we present the signal accumulation scheme adopting multi-channel information. To provide a more specific, clear, and understandable explanation, this study adopts the widely-used Texas Instruments (TI) AWR1642 millimeter-wave radar system as a representative example, and our algorithm is universally applicable to similar multi-antenna systems.

**Figure 3:**
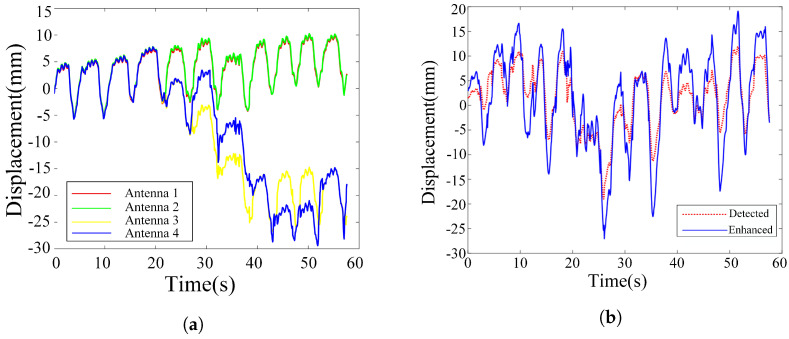
(**a**) Phase signals from four channels. (**b**) Original phase signal (at the detection range bin) and enhanced signal after accumulation.

Most radar-based spatial domain systems still rely on single-channel processing. Although some studies (e.g., [19]) use direct summation of multi-channel signals (referred to here as equal gain combination (EGC)), this approach often underperforms due to channel variability. For instance, as shown in Figure 3a, signals obtained from four antennas exhibit noticeable differences: the waveforms from antennas 3 and 4 deviate substantially in shape from those of antennas 1 and 2, thereby rendering simple signal summation ineffective.

Spatial domain processing faces several key challenges: vital sign signals vary across channels, resulting in low correlation [17], and phase unwrapping is nonlinear and complex [37]. As a result, direct multi-channel summation often fails to deliver optimal performance. To address the limitations of the EGC scheme, we propose a spatial domain coherent accumulation (SDCA) method that follows the “fewer but better” principle. Instead of using all channels, SDCA adaptively selects a small number of high-quality channels, thereby influencing accumulation gain and signal quality.

During heartbeat signal detection, the entire radar data frame is partitioned into *n* distinct groups, with each group containing *J* frames. Based on empirical analysis, we set J=128. Within each group, a reference range bin is selected from the *J* frames. Additionally, for each frame, the range bin with the maximum energy across all channels is dynamically selected as the reference. The specific processing steps for each group of data are outlined as follows.

The selection of the range bin is performed first, where the energy of the phase signals from the four channels is calculated as:

(6) $$E_{ki}=\sum_{j=1}^{J}x_{ki}^{2}\left(j\right)$$

where $E_{ki}$ denotes the signal energy of the *i*th range bin in the *k*th channel, $x_{ki}$ represents the phase signal, and *J* is the total number of frames in each group. The reference channel and reference range bin are then selected according to the maximum energy criterion:

(7) $$\left(k_{0},i_{0}\right)=\underset{k_{0}\in \left\{1,2,3,4\right\}}{\arg\max}\;E_{k_{0}i_{0}},1\leq i_{0}\leq 256,i_{0}\in Z$$

where $i_{0}$ is the chosen reference range bin, and $k_{0}$ is the corresponding reference channel. Since the AWR1642 radar is equipped with four antennas, the antenna index $k_{0}$ ranges from 1 to 4. A 256-point FFT is performed along the fast-time dimension, so the range bin index $i_{0}$ spans from 1 to 256. Both the reference channel and range bin are selected once per group, i.e., once every *J* frames.

After determining the reference channel, an optimal channel for signal accumulation is selected from the remaining three receiving channels of the AWR1642 radar. Following the approach in [10], the optimal channel is chosen based on the correlation coefficient between the reference channel and each candidate channel. Specifically, let ${\hat{\phi }}_{k_{0}}$ and ${\hat{\phi }}_{o}$ denote the phase signal vectors obtained after phase unwrapping from the reference channel and another channel, respectively. The Pearson correlation coefficient between these two vectors is then computed as:

(8) $$\rho \left({\hat{\phi }}_{k_{0}},{\hat{\phi }}_{o}\right)=\frac{\mathrm{cov}({\hat{\phi }}_{k_{0}},{\hat{\phi }}_{o})}{\sigma_{{\hat{\phi }}_{k_{0}}}\sigma_{{\hat{\phi }}_{o}}}$$

where $\mathrm{cov}({\hat{\phi }}_{k_{0}},{\hat{\phi }}_{o})$ represents the covariance of these vectors, $\sigma_{{\hat{\phi }}_{k_{0}}}$ and $\sigma_{{\hat{\phi }}_{o}}$ are the corresponding standard deviations, respectively. Secondly, we select the channel with the highest correlation coefficient among those exceeding the predetermined threshold, which is calculated by:

(9) $$l=\arg\max\;\rho_{k},\;\;1\leq k\leq 4,\;k\neq k_{0}$$

where $\rho_{k}$ denotes the correlation coefficient between the *k*th channel and the reference channel $k_{0}$, *l* represents the index of the channel corresponding to the maximum correlation coefficient. Finally, we evaluate whether the correlation coefficient $\rho_{l}$ exceeds the predefined threshold γ. If $\rho_{l}\geq \gamma$, we accumulate the phase signal by summing phase signals of these two channels directly (channel *k* and $k_{0}$ ). Otherwise, we double the phase signal of $k_{0}$. This selection process can be expressed as:

(10) $$\phi \left(j\right)=\left\{\begin{array}{c} \rho_{l}\geq \gamma ,\;\phi_{k_{0}}\left(j\right)+\phi_{l}\left(j\right),\;l\neq k_{0}\\ \rho_{l}<\gamma ,\;\phi_{k_{0}}\left(j\right)\times 2 \end{array}\right.$$

where $\phi_{k_{0}}$ and $\phi_{l}$ represent the phase signals of the reference channel and the optimal channel, respectively. Based on extensive simulations, we set the threshold γ=0.8 in our experiments. Channels with correlation coefficients above 0.8 are selected for accumulation, as this threshold consistently provides stable and effective performance. A lower threshold such as 0.6 may introduce less correlated channels, leading to interference, while a higher threshold like 0.9 may exclude too many channels, weakening the benefit of multi-channel accumulation. Therefore, γ=0.8 offers a reasonable trade-off between selectivity and accumulation effectiveness. Figure 3b compares the original signal and the enhanced signal after applying SDCA, demonstrating improved displacement amplitude via spatial accumulation.

SDCA leverages multi-antenna data to enhance signal quality through optimal channel selection. However, since human reflections span multiple adjacent range bins [20], by extending the SDCA approach to incorporate signals from these distributed range bins [25], further improvements in HR detection performance can be expected.

Although [20] proposed selecting adjacent bins with high correlation (e.g., Pearson coefficient >0.8), we observe that, after spatial accumulation, the correlation between bins frequently decreases, limiting the applicability of this method. Similarly, refs. [21,22] explored multi-bin or region accumulation, but little gain can be achieved or computational burden is added when combined with SDCA.

To address this, we propose a simple yet effective method: After selecting the reference bin, only the adjacent bin (either preceding or following) with the highest energy is used for accumulation, ensuring improved performance with minimal complexity.

The following sections introduce the MDCA proposed in this study:

1. Spatial domain processing: First, select the channel $k_{0}$ with the highest energy as the reference channel. Then, identify the channel $k_{i}$ that has the highest correlation with $k_{0}$ as the optimal channel. If the correlation coefficient exceeds a predefined threshold, the phase signals from these two channels are summed to perform SDCA.
2. Temporal domain processing: Determine the range bin $i_{0}$ with the highest energy as the reference bin. Then, select the adjacent bin $i_{1}$ that has the next highest energy relative to $i_{0}$. For each of these two bins, apply the spatial domain accumulation described above.
3. Joint temporal–spatial accumulation: Finally, sum the signals from the two selected range bins (after spatial domain accumulation) to complete the joint accumulation across both the temporal and spatial domains.

By processing each data group using the above method, the proposed MDCA approach can be effectively implemented. This method fully leverages both spatial and temporal information for signal accumulation, thereby significantly improving the accuracy of heart rate estimation.

**Figure 4:**
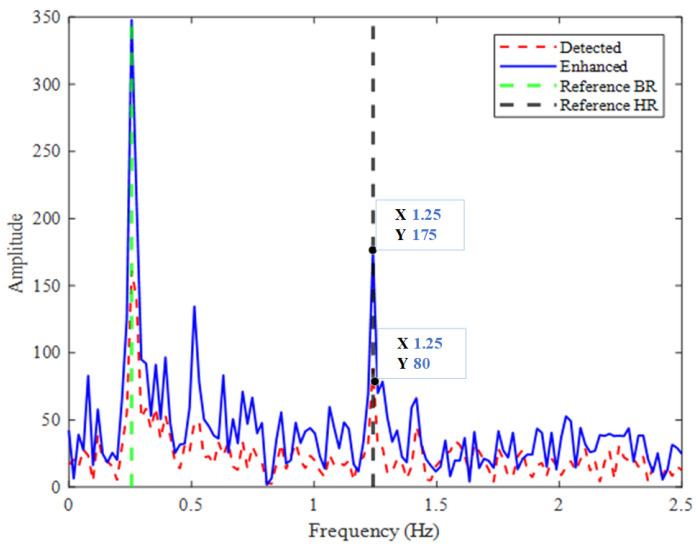
Spectrum of original and enhanced signals.

Figure 4 illustrates the frequency spectra of the single-channel signal and the signal after MDCA enhancement. The red dashed line represents the single-channel method, the blue solid line corresponds to the MDCA method proposed in this study, the green dashed line marks the reference respiration rate, and the black dashed line indicates the reference heart rate. It can be observed that the main spectral peaks remain located at the reference respiratory and heart rates after enhancement. Meanwhile, the blue line exhibits significantly higher amplitude than the red dashed line, indicating that the vital sign components are effectively enhanced by MDCA.

To quantify the enhancement effect, we also calculated the frequency-domain SNR, defined as the ratio between the spectral peak amplitude at the target frequency (heartbeat) and the average noise amplitude in neighboring bins, excluding known harmonics. The specific formula is provided in (11), and follows conventions in radar-based vital sign studies:

(11) $$\begin{array}{cc} \; & SNR=10\mathrm{lg}((\sum_{k=k_{\mathrm{peak}}-2}^{k_{\mathrm{peak}}+2}{\left|X\left(k\right)\right|}^{2})/(P-\sum_{k=k_{\mathrm{peak}}-2}^{k_{\mathrm{peak}}+2}{\left|X\left(k\right)\right|}^{2})) \end{array}$$

where X(k) is a Discrete Fourier Transform (DFT) of signal x(n), $P=\sum_{k=k_{1}}^{k_{2}}{\left|X\left(k\right)\right|}^{2}$ is the signal power, $k_{1}=\mathrm{int}\left[0.8/(f_{s}/N)\right]$, $k_{2}=\mathrm{int}\left[2/(f_{s}/N)\right]$, $k_{peak}$ is the maximum amplitude position within the frequency range of 0.8–2 Hz, $f_{s}$ is the sampling frequency, and *N* is the number of sampling points. In the example of Figure 4, the unenhanced signal has an SNR of −7.27 dB, whereas the MDCA-enhanced signal achieves −2.60 dB, corresponding to a notable improvement of 4.67 dB.

**Figure 5:**
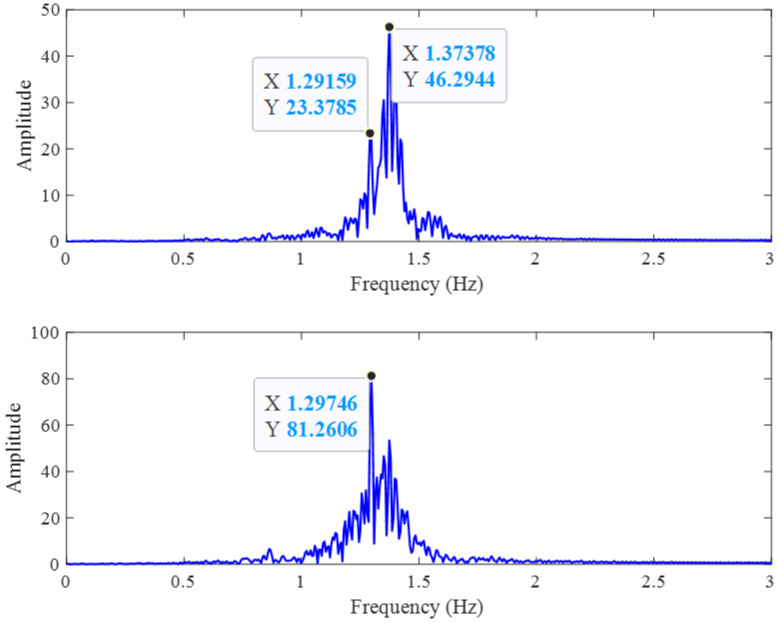
Spectrum of heartbeat signals without (**top**) and with (**bottom**) MDCA.

To further assess the effectiveness of MDCA, a more detailed spectral analysis was performed. Although the true frequency components remain unchanged, we observed that in some cases, spurious peaks in the unprocessed signal can be stronger than the actual physiological peaks, potentially leading to incorrect frequency estimation. As illustrated in Figure 5, with a reference heart rate of 78 bpm (approximately 1.3 Hz), the single-channel signal exhibits a stronger peak near 1.37 Hz, resulting in a deviation of 0.07 Hz (approximately 4.2 bpm). After applying MDCA, the heart rate component is significantly enhanced through accumulation and becomes the dominant peak, thereby improving the estimation accuracy.

Consequently, the SNR is substantially improved by MDCA, which provides a stronger foundation for subsequent precise frequency estimation.

### 3.2. Phase Extraction and Signal Separation

#### 3.2.1. Phase Unwrapping and Phase Difference Computation

In FMCW radar-based vital sign detection, the tiny chest wall movements induced by respiration and heartbeat lead to phase variations in the received signals. To extract these small displacements, we focus on the phase information of the baseband signal after range FFT.

Let $S_{n}\left(t\right)$ represent the complex baseband signal of the *n*th receiving channel at slow time *t*. The raw phase is given by $\phi_{n}\left(t\right)=\arg\left(S_{n}\left(t\right)\right)$. However, due to the periodic nature of the arctangent function, phase discontinuities (jumps between −π and π) can occur. Therefore, we apply phase unwrapping along the slow-time dimension to recover the continuous phase trajectory:

(12) $${\tilde{\phi }}_{n}\left(t\right)=\mathrm{unwrap}\left(\arg\left(S_{n}\left(t\right)\right)\right)$$

The specific procedure is as follows: whenever the difference between two consecutive phases exceeds −π or π, a 2π increment or decrement is applied to the latter phase to eliminate the jump, thereby unwrapping the phase into a continuous trajectory. After obtaining the unwrapped phase, we compute the inter-frame phase difference to eliminate static components and separate the dynamic motions caused by vital signs:

(13) $$\Delta \phi_{n}\left(t\right)={\tilde{\phi }}_{n}\left(t\right)-{\tilde{\phi }}_{n}\left(t-1\right)$$

This differential phase signal $\Delta \phi_{n}\left(t\right)$ captures the micro-motions due to chest displacement and is the input for the subsequent signal enhancement and frequency estimation steps.

#### 3.2.2. Variational Mode Decomposition Algorithm

VMD serves as a critical signal processing technique in our framework for accurate heartbeat signal extraction and interference suppression. As a non-recursive and adaptive decomposition method, VMD effectively separates an input signal f(t) into *K* distinct Intrinsic Mode Functions (IMFs) $u_{k}\left(k=1,2,3,\ldots ,K\right)$ [38]. Each extracted IMF component exhibits specific spectral properties, maintaining a certain bandwidth around its central frequency $\omega_{k}$. This decomposition process can be formulated as an optimization problem aiming to minimize the sum of the bandwidths for each mode, which can be expressed as:

(14) $$\min_{\left\{u_{k}\right\},\left\{\omega_{k}\right\}}\left\{\sum_{k}{∥\partial_{t}\left[\left(\sigma \left(t\right)+\frac{j}{\pi t}\right)*u_{k}\left(t\right)\right]e^{-j\omega_{k}t}∥}_{2}^{2}\right\}\;s.t.\;\sum_{k}u_{k}\left(t\right)=f$$

where $\left\{u_{k}\right\}=\left\{u_{1},\ldots ,u_{K}\right\}$ is the set of *K* IMF components, and $\left\{\omega_{k}\right\}=\left\{\omega_{1},\ldots ,\omega_{K}\right\}$ represents their corresponding central frequencies. The constrained variational problem (14) is solved by introducing a quadratic penalty parameter and a Lagrange multiplier λ(t). The augmented Lagrange function is expressed as:

(15) $$\begin{array}{cc} L\left(\left\{u_{k}\right\},\left\{\omega_{k}\right\},\lambda \right) & =\alpha \sum_{k}{∥\partial_{t}\left[\left(\sigma \left(t\right)+\frac{j}{\pi t}\right)*u_{k}\left(t\right)\right]e^{-j\omega_{k}t}∥}_{2}^{2}+{∥f\left(t\right)-\sum_{k}u_{k}\left(t\right)∥}_{2}^{2}\\ \; & +\langle \lambda \left(t\right),f\left(t\right)-\sum_{k}u_{k}\left(t\right)\rangle \end{array}$$

The Alternating Direction Method of Multipliers (ADMM) is used to find the saddle point of the augmented Lagrangian. The IMFs are updated in the frequency domain using a Wiener filter:

(16) $${\hat{u}}_{k}^{n+1}\left(\omega \right)=\frac{\hat{f}\left(\omega \right)-\sum_{i\neq k}{\hat{u}}_{i}\left(\omega \right)+\frac{\hat{\lambda }\left(\omega \right)}{2}}{1+2\alpha {\left(\omega -\omega_{k}\right)}^{2}}$$

where ${\hat{u}}_{k}^{n+1}\left(\omega \right)$, $\hat{f}\left(\omega \right)$ and $\hat{\lambda }\left(\omega \right)$ denote the Fourier transforms of $u_{k}^{n+1}$, f(t), and λ(t), respectively. The central frequency $\omega_{k}^{n+1}$ is updated according to (17), which corresponds to the IMF power spectrum:

(17) $$\omega_{k}^{n+1}=\frac{\int_{0}^{\infty }\omega {\left|{\hat{u}}_{k}\left(\omega \right)\right|}^{2}d\omega }{\int_{0}^{\infty }{\left|{\hat{u}}_{k}\left(\omega \right)\right|}^{2}d\omega }$$

The iteration termination condition is as follows:

(18) $$\sum_{k}\frac{{∥{\hat{u}}_{k}^{n+1}-{\hat{u}}_{k}^{n}∥}_{2}^{2}}{{∥{\hat{u}}_{k}^{n}∥}_{2}^{2}}\leq \varepsilon$$

The specific iterative procedure of VMD is as follows:

(1) Manually select appropriate mode decomposition number *K* and penalty factor α.

(2) Initialize $\left\{{\hat{u}}_{k}^{1}\right\}$, $\left\{{\hat{\omega }}_{k}^{1}\right\}$, ${\hat{\lambda }}^{1}$ and *n*.

(3) For k=1,2,…,K, update ${\hat{u}}_{k}^{n+1}$ by using (16).

(4) For k=1,2,…,K, update $\omega_{k}^{n+1}$ by using (17).

(5) Update ${\hat{\lambda }}^{n+1}\left(\omega \right)$ by using the following formula:

(19) $${\hat{\lambda }}^{n+1}\left(\omega \right)={\hat{\lambda }}^{n}\left(\omega \right)+\tau \left(\hat{f}\left(\omega \right)-\sum_{k}{\hat{u}}_{k}^{n+1}\left(\omega \right)\right)$$

(6) Repeat steps (3) to (5) until the iteration termination condition (18) is satisfied to end the loop. Then, obtain each ${\hat{u}}_{k}$ and center frequency $\omega_{k}$. Finally, each IMF component $u_{k}\left(t\right)$ is obtained through the inverse Fourier transform.

### 3.3. Spectrum Analysis Spectrum

The fundamental component of the HR signal is often closely spaced in frequency with respiratory harmonics, cross-modulation components, and other physiological or environmental interferences. In many cases, the frequency separation between the HR fundamental and nearby interference is very narrow, which poses a significant challenge for conventional spectral analysis methods such as FFT. These methods typically suffer from limited resolution, making it difficult to distinguish and accurately extract the true HR component. Therefore, a frequency estimation method with high resolution, high accuracy, and low computational complexity is essential for robust and reliable heart rate detection. Although the MUSIC and estimation of signal parameters via rotational invariance techniques (ESPRIT) algorithms have super-resolution capability, it is difficult to adopt them for HR detection due to their massive computational requirements. Consequently, an algorithm featuring super-resolution capability, high spectral analysis precision, and low computational complexity is imperative for achieving accurate and efficient heart rate estimation.

To overcome the aforementioned challenges, we adopt the FIIB algorithm for HR estimation. FIIB, originally developed for direction-of-arrival (DOA) estimation in the spatial domain, effectively combines super-resolution capability with low computational complexity, making it particularly suitable for radar-based physiological monitoring.

Following the approach in [39], we employ an iterative “estimate–subtract” strategy, where a coarse estimation stage first identifies dominant frequency components using the FFT, and a fine estimation stage then iteratively refines these results to minimize estimation bias and improve resolution. The complete workflow of the FIIB algorithm is summarized in Algorithm 1.
**Algorithm 1** Frequency Estimation Based on FIIB1:**Initialization:**2:**for** l=1 to *L* **do**3:    ${\hat{f}}_{l}\leftarrow 0$4:    ${\hat{A}}_{l}\leftarrow 0$5:**end for**6:q←07:X←FFT(x,N)8:**while** q<Q and not converged **do**9:    **for** l=1 to *L* **do**10:        **if** q=0 **then**                ▹ Coarse Estimate11:           **for** k=0 to N−1 **do**12:               $\hat{X}\left(k\right)\leftarrow X\left(k\right)-\sum_{\begin{array}{c} i=1\;i\neq l \end{array}}^{L}{\hat{A}}_{i}{\hat{S}}_{i}\left(k\right)$13:           **end for**14:           ${\hat{m}}_{l}\leftarrow \underset{0\leq k\leq N-1}{\arg\;\max}{\left|\tilde{X}\left(k\right)\right|}^{2}$15:           ${\hat{f}}_{l}\leftarrow {\hat{m}}_{l}/N$16:        **else**                   ▹ Accurate Estimate17:           **for** $p\in \left\{-\frac{1}{2},\frac{1}{2}\right\}$ **do**18:               $X_{p}\left({\hat{f}}_{l}\right)\leftarrow X\left({\hat{f}}_{l}+\frac{p}{N}\right)$19:               ${\tilde{X}}_{p}\left({\hat{f}}_{l}\right)\leftarrow X_{p}\left({\hat{f}}_{l}\right)-\sum_{\begin{array}{c} i=1\;i\neq l \end{array}}^{L}{\hat{A}}_{i}{\hat{S}}_{i}\left({\hat{f}}_{l}+\frac{p}{N}\right)$20:           **end for**21:           $\delta \leftarrow \frac{1}{2}\cdot ℜ\left[\frac{{\tilde{X}}_{0.5}\left({\hat{f}}_{l}\right)+{\tilde{X}}_{-0.5}\left({\hat{f}}_{l}\right)}{{\tilde{X}}_{0.5}\left({\hat{f}}_{l}\right)-{\tilde{X}}_{-0.5}\left({\hat{f}}_{l}\right)}\right]$22:           ${\hat{f}}_{l}\leftarrow {\hat{f}}_{l}+\delta /N$23:        **end if**24:        ${\hat{A}}_{l}\leftarrow \frac{1}{N}\left[\sum_{k=0}^{N-1}x\left(k\right)e^{-j\frac{2\pi }{N}k{\hat{f}}_{l}}-\sum_{\begin{array}{c} i=1\;i\neq l \end{array}}^{L}{\hat{A}}_{i}{\hat{S}}_{i}\left({\hat{f}}_{l}\right)\right]$25:    **end for**26:    q←q+127:**end while**28:**Output:** $\left\{{\hat{f}}_{l},{\hat{A}}_{l}\right\}$ for l=1,2,…,L

Specifically, for the *l*th source, we first subtract the previously estimated source in line 12, then locate the highest peak of the spectrum in line 14. This can reduce the impact of other signals on the current signal (*l*th signal). Note that in line 12, ${\hat{S}}_{i}\left(k\right)$ represents the DFT coefficient of the *i*th frequency component at the normalized frequency f=k/N. This relationship is formally expressed as:

(20) $${\hat{S}}_{i}\left(k\right)={\hat{S}}_{i}\left(f\right){|}_{f=k/N}$$

where ${\hat{S}}_{i}\left(f\right)$ denotes Fourier transform of *i*th frequency component which can be expressed as:

(21) $${\hat{S}}_{i}\left(f\right)=\sum_{k=0}^{N-1}e^{j2\pi k{\hat{f}}_{i}}e^{-j2\pi f}=\frac{1-e^{j2\pi N({\hat{f}}_{i}-f)}}{1-e^{j2\pi ({\hat{f}}_{i}-f)}}$$

The normalized coarse estimate obtained in line 15 serves as input for spectral refinement in line 19 to line 22, where Fourier coefficients interpolation techniques are applied to enhance estimation accuracy, achieving the fine estimation [40].

To refine the measurement range, line 19 calculates the DFT coefficients ${\tilde{X}}_{p}\left({\hat{f}}_{l}\right)$ of complex exponential signal at frequency ${\hat{f}}_{l}$.

These coefficients are subsequently subtracted from the spectral leakage components associated with other estimated frequency points, which are expressed as:

(22) $${\tilde{X}}_{p}\left({\hat{f}}_{l}\right)={\tilde{X}}_{p}\left({\hat{f}}_{l}+\frac{p}{N}\right)=X_{p}\left({\hat{f}}_{l}+\frac{p}{N}\right)-\sum_{i=1\;i\neq l}^{L}A_{i}{\hat{S}}_{i}\left({\hat{f}}_{l}+\frac{p}{N}\right)$$

where the leakage DFT coefficients can be expressed as:

(23) $${\hat{S}}_{i}\left({\hat{f}}_{l}+\frac{p}{N}\right)=\frac{1+e^{j2\pi N({\hat{f}}_{i}-{\hat{f}}_{l})}}{1-e^{j2\pi ({\hat{f}}_{i}-{\hat{f}}_{l}-\frac{p}{N})}}$$

Subsequently, spectral interpolation is performed using the leakage-compensated DFT coefficients ${\tilde{X}}_{p}\left({\hat{f}}_{l}\right)$ to estimate the frequency offset. Let δ denote the true frequency deviation between the *l*th complex exponential signal and the maximum spectral line obtained from coarse estimation, where δ∈[−0.5,0.5]. The frequency offset of the complex exponential signal is then given by δ/N, and the spectral estimation result for the q+1th iteration is expressed as:

(24) $$f_{l}=\frac{{\hat{m}}_{l}+\delta }{N}={\hat{f}}_{l}+\frac{\delta }{N}$$

Finally, the frequency estimate of the *l*th complex exponential component is refined by incorporating the offset δ. The associated complex amplitude is then calculated by compensating for spectral leakage from other components at this frequency. The estimation proceeds sequentially from the strongest to the weakest signal, and this iterative process is repeated for all components over *Q* iterations.

The FIIB algorithm requires only a single FFT computation and combines a small number of Fourier coefficients with interpolation techniques, thereby effectively reducing the computational complexity.

#### 3.3.1. Frequency Estimation Performance Evaluation

To analyze the frequency estimation performance of FIIB, we considered a simulation scenario involving dual-frequency signals defined as:

(25) $$x\left(n\right)=a_{1}\cos\left(2\pi f_{1}nT_{s}\right)+a_{2}\cos\left(2\pi f_{2}nT_{s}\right)$$

where $T_{s}=1/f_{s}$ is the sampling period, $a_{1}$ and $a_{2}$ are the amplitudes of the two signals. $f_{1}$ and $f_{2}$ denote the frequencies of the two signals. The sampling frequency is set to $f_{s}=20$ Hz, and the number of samples N=200. The noise is additive white Gaussian noise (AWGN), and the SNR varies from −15 dB to 10 dB in steps of 1 dB. In all examples, Monte Carlo experiments were performed independently for 5000 times, and the performance of the FIIB method was compared with that of the FFT and MUSIC algorithms. The mean squared error (MSE) was used in the experiments to evaluate the accuracy of the frequency estimation, defined as:

(26) $$MSE=\frac{1}{M}\sum_{i=1}^{M}{\left({\hat{f}}_{i}-f_{r}\right)}^{2}$$

where ${\hat{f}}_{i}$ is the estimated frequency, *M* denotes the number of Monte Carlo trials, which is set to 5000 in this study and $f_{r}$ is the real frequency. The simulation parameters are set according to the actual conditions of radar monitoring of HR, $f_{1}=1.05\;\mathrm{Hz}$, $f_{2}=f_{1}+1.5\Delta f$, $a_{1}=0.2$, $a_{2}=0.15$, and Δf is the frequency resolution of FFT, which can be written as:

(27) $$\Delta f=\frac{f_{s}}{N}$$

**Figure 6:**
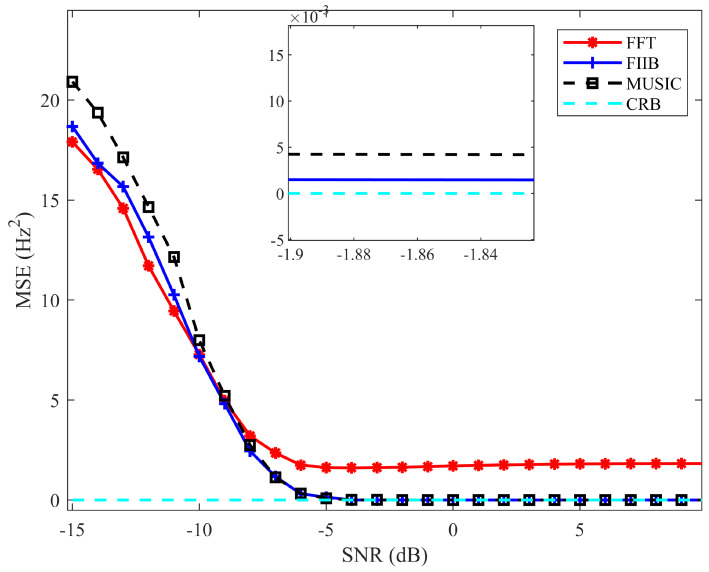
Overall performance comparison of three frequency estimation methods.

Figure 6 presents the MSE curves plotted against the SNR for the three algorithms. The results demonstrate that the FIIB algorithm approaches the Cramér-Rao Bound (CRB) more closely when the SNR exceeds −6 dB. While the MUSIC algorithm achieves comparable measurement accuracy to FIIB, it does so at the expense of significantly higher computational complexity. As a result, the FIIB algorithm offers superior spectral analysis performance combined with low computational cost, rendering it particularly well-suited for heart rate detection applications.

#### 3.3.2. Resolution and Computational Complexity Comparison

**Figure 7:**
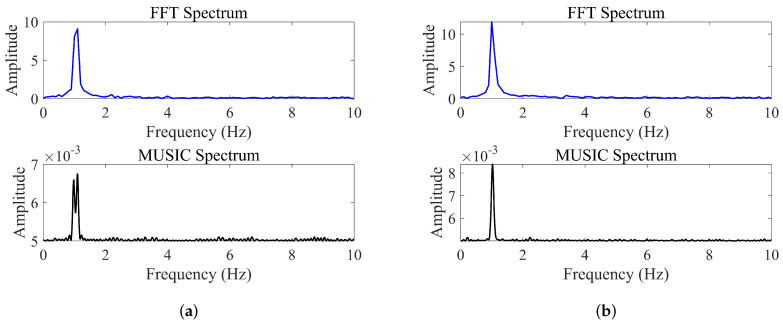
Frequency spectra comparison of three methods at different resolutions. (**a**) 0.75× FFT resolution. (**b**) 0.5× FFT resolution.

Figure 7a,b compares the performance of MUSIC and FFT algorithms at an SNR of 20 dB, using frequency intervals set at 0.75 and 0.5 times the FFT resolution, respectively. FFT fails to resolve the closely spaced frequencies in both scenarios due to its limited resolution. In contrast, MUSIC successfully resolves the 0.75 times interval but fails at the 0.5 times interval. Notably, FIIB employs an efficient ‘estimate–subtract’ approach, which does not directly produce a conventional spectral plot. As indicated in Table 1, FIIB accurately resolves targets at both 0.75 and 0.5 times the FFT resolution, with a RMSE—defined as the square root of the MSE—below 0.0026 Hz, demonstrating its superior super-resolution capability and estimation accuracy.

Further analysis reveals that even at a frequency spacing of 0.8 times the FFT resolution, the FFT method fails to resolve two closely spaced frequency components. The MUSIC algorithm successfully resolves signals starting at 0.7 times the FFT resolution, whereas the proposed FIIB algorithm consistently resolves the two targets across all tested intervals ranging from 0.4 to 0.8 times the FFT resolution.

Additionally, we compared the computational complexity of FFT, MUSIC, and FIIB algorithms on a laptop equipped with an Intel i5-8265U CPU and 8 GB RAM. Their execution times are approximately 0.001 s, 26 s, and 0.06 s, respectively. MUSIC is significantly more computationally demanding than the other two algorithms, primarily due to the eigenvalue decomposition involved. While FFT is the fastest, it lacks super-resolution capabilities and suffers from lower accuracy. FIIB provides a favorable trade-off, combining high accuracy and super-resolution with low computational complexity, rendering it well-suited for real-time heart rate measurement applications.

## 4. Experiments

Experiments were conducted using a TI AWR 1642 mm-wave FMCW radar system equipped with 2 transmitters (TX) and 4 receivers (RX). Data were transferred via USB and processed on a laptop using MATLAB R2021b (MathWorks, Natick, MA, USA). The radar settings are detailed in Table 2. The system operated at a center frequency of 79 GHz with a bandwidth of 3.99 GHz.

**Figure 8:**
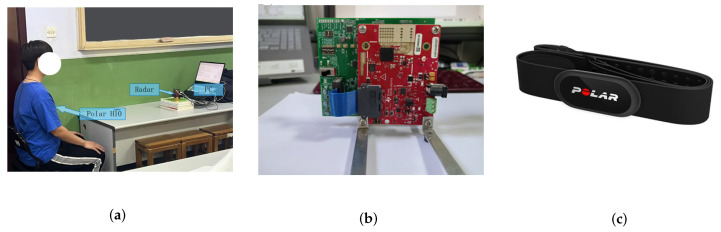
Demonstration of the experimental setup and equipment. (**a**) Measurement environment. (**b**) Radar hardware. (**c**) Reference chest sensor.

All tests were conducted in an empty laboratory where subjects sat motionless facing the radar. A Polar H10 heart rate sensor was worn by the subjects to serve as the reference measurement (see Figure 8a).

A total of 1200 frames were collected at a sampling rate of 20 Hz. Data processing employed sliding windows of 26 s duration with a 25-s overlap, resulting in update intervals of 1 s.

Moreover, the radar system and Polar H10 sensor were synchronized to ensure temporal alignment. The RMSE metric was employed to quantify the heart rate estimation accuracy, defined as:

(28) $$RMSE=\sqrt{\frac{1}{L}\sum_{i=1}^{L}{\left({\hat{f}}_{i}-f_{r}\right)}^{2}}$$

where *L* denotes the total number of sliding windows.

Figure 8b,c depict the experimental setup and equipment.

### 4.1. Measurement Results

**Figure 9:**
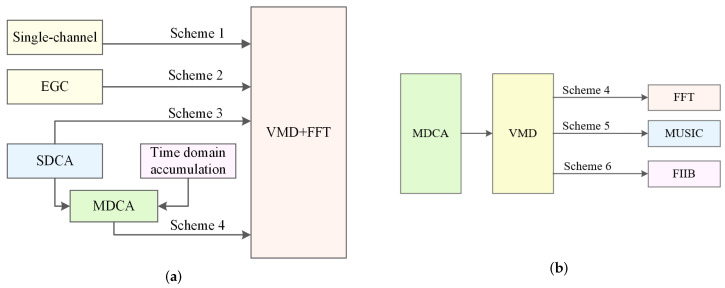
(**a**) Brief flow diagram of four schemes. (**b**) Brief flow diagram of three schemes.

To validate the effectiveness of SDCA and MDCA, VMD was employed for heartbeat extraction, and FFT was used for heart rate estimation, while keeping all other processing steps consistent. Twenty volunteers participated in tests under four different processing schemes: (1) Single-channel processing without accumulation. (2) EGC, which sums all channels. (3) SDCA, performing spatial domain accumulation. (4) MDCA, combining both spatial and temporal accumulation. These four schemes are illustrated in Figure 9a, and the corresponding results are summarized in Table 3.

As presented in Table 3, the average RMSE values for the four schemes were 4.30, 3.72, 2.60, and 2.20 bpm, respectively. All accumulation schemes outperform the single-channel method. SDCA (Scheme 3) achieved an RMSE improvement of 1.12 bpm compared to EGC (Scheme 2), attributed to its optimal channel selection. MDCA (Scheme 4) further reduced the RMSE by 0.4 bpm, yielding a total improvement of 2.1 bpm over the single-channel scheme and 1.52 bpm compared to EGC. Notably, Group 13 in Table 3 exhibits two relatively large errors. Scheme 1 does not incorporate any signal accumulation, which results in a low SNR for certain subjects, thus affecting frequency estimation accuracy. In addition, Scheme 2’s EGC approach performs poorly when channel amplitudes vary significantly.

**Figure 10:**
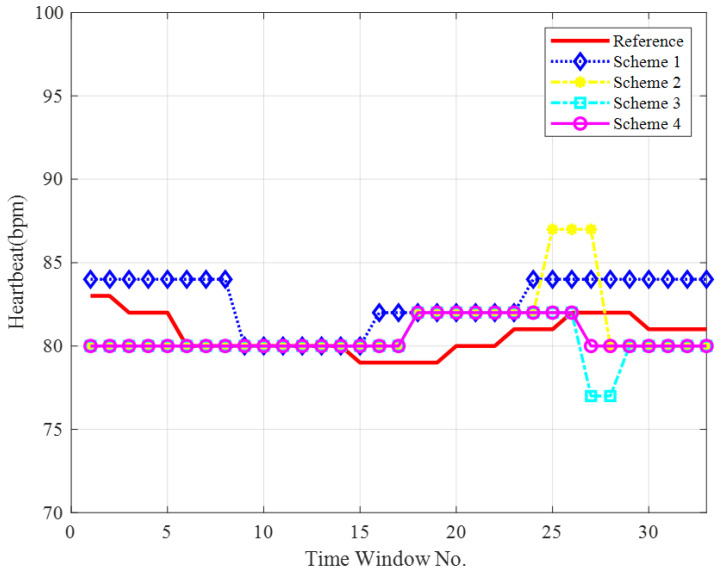
HR curves under different schemes.

To visually compare the performance of the four HR detection schemes, representative experimental data from Table 3 were selected, and the corresponding HR waveforms were plotted in Figure 10.

Obviously, the MDCA method, represented by the pink curve, aligns more closely with the actual reference HR curve. While both the SDCA (cyan curve) and EGC (yellow curve) show general agreement with the reference HR profile, noticeable fluctuations occur between 24 s and 29 s, causing deviations in the final measurements. The single-channel method, represented by the blue curve, exhibits significant errors compared to the baseline throughout most time intervals. These observations indicate that MDCA can significantly improve the accuracy of HR detection.

To further validate the performance gains provided by the FIIB algorithm, we conducted comparative experiments. As illustrated in Figure 9b, all other processing conditions were kept identical—specifically, the MDCA method for preprocessing and the VMD method for heartbeat signal extraction. The only difference lay in the spectrum analysis algorithm: the HR of 20 volunteers was estimated using FFT in Scheme 4, MUSIC in Scheme 5, and FIIB in Scheme 6, respectively. It is worth noting that Scheme 6 represents the complete scheme proposed in this study, as illustrated in Figure 2. The experimental results are summarized in Table 4.

The average RMSE values for FFT, MUSIC, and FIIB were 2.20 bpm, 1.28 bpm, and 1.12 bpm, respectively. Benefiting from its high resolution and two-stage estimation process, FIIB outperformed FFT and MUSIC by 1.08 bpm and 0.16 bpm, respectively. Moreover, FIIB demonstrates lower computational complexity and higher efficiency compared to MUSIC, rendering it highly suitable for real-time heart rate monitoring.

**Figure 11:**
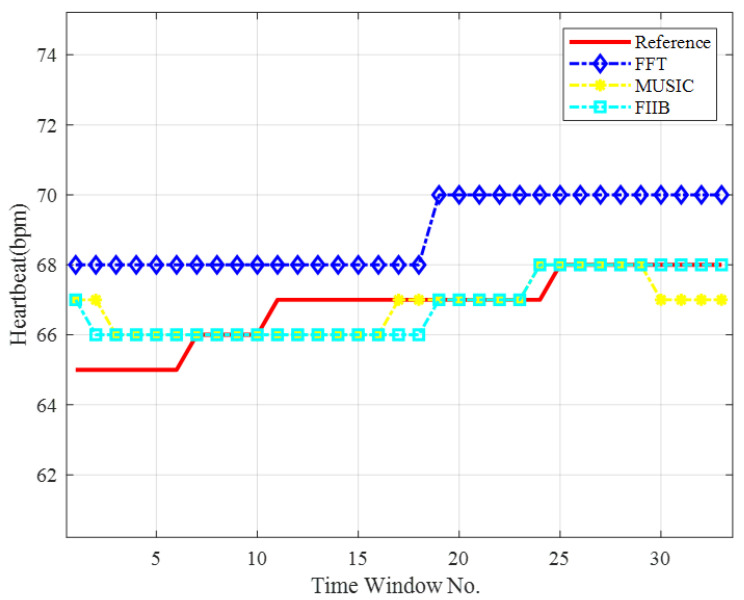
HR curves for different frequency measurement methods.

A representative dataset was selected from Table 4, and the corresponding heart rate curves are depicted in Figure 11. As shown, the HR estimates obtained using FIIB and MUSIC closely follow the reference values, accurately capturing the fluctuations in heart rate. In contrast, the FFT-based estimates exhibit significant deviations from the reference during the 1st to 10th and 18th to 33rd sliding windows.

### 4.2. FIIB Parameter Selection and Reordering Strategy for HR Detection

The number of effective frequency components *L* and the number of iterations *Q* are two critical parameters influencing the performance of the FIIB algorithm. Notably, existing studies have not yet established standardized guidelines for parameter selection in vital signal detection applications. This section addresses three fundamental issues regarding the practical implementation of FIIB for heart rate detection: (1) determination of the effective frequency components *L*, (2) selection of the number of iterations *Q*, and (3) signal reordering strategy.

#### 4.2.1. Determination of the Number of Effective Frequency Components *L*

To evaluate the effect of the parameter *L* (i.e., the number of frequency components) on the heart rate estimation accuracy of the FIIB algorithm, a series of experiments were conducted in which all other parameters were held constant while varying *L*. A total of 200 experimental trials were performed, and the average RMSE values calculated over every 20 experiments are summarized in Table 5.

The experimental results show that the best heart rate estimation accuracy, reflected by the lowest RMSE, is achieved when L=4. Although setting L=2 yields only slightly worse results, this is mainly attributed to the insufficient number of components considered. In such cases, leakage from dominant interfering frequencies is not effectively suppressed, which adversely affects the extraction of the heartbeat signal. On the other hand, increasing *L* to 6 or 8 causes the algorithm to estimate additional frequency components, potentially introducing unnecessary complexity. Therefore, L=4 is recommended as it provides a balanced trade-off between estimation accuracy and computational efficiency.

#### 4.2.2. Selection of the Number of Iterations *Q*

To determine the optimal number of iterations *Q*, we recorded the HR estimates at each iteration of the FIIB algorithm and evaluated whether the predefined stopping criterion was satisfied, which can be expressed as:

(29) $$\left|f_{k}-f_{k-1}\right|<\varepsilon$$

where $f_{k}$ is the estimated HR after the *k*th iteration, $f_{k-1}$ is the estimated HR after the k−1th iteration, and ε is a very small threshold (ε=0.001).

**Figure 12:**
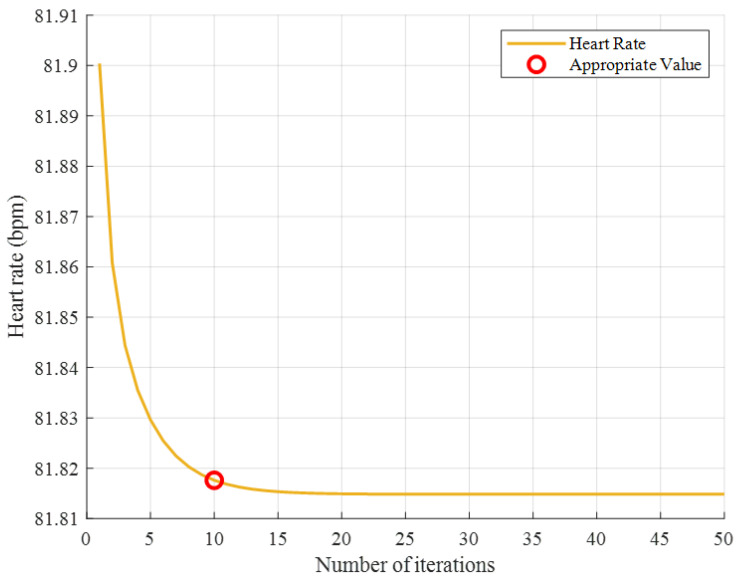
Estimated change curve of HR.

Through extensive simulation experiments, we observed consistent trends in the results. Figure 12 presents a representative example illustrating how the estimated HR values vary with the number of iterations.

The true value in this experiment is 81.81 bpm. As shown in Figure 12, after approximately 10 iterations, the estimated values gradually stabilize. In fact, 5 iterations can also meet the accuracy requirements of the experiment, but 10 iterations would be more precise. Based on the results of extensive simulation experiments, we recommend Q = 10.

#### 4.2.3. Signal Reordering Strategy

During the frequency estimation process, spectral leakage occurs when the target frequency does not align with the discrete frequency bins of the DFT, resulting in the dispersion of signal energy across adjacent bins and potentially shifting the main lobe peak away from the true frequency. In the coarse estimation stage of the FIIB algorithm, FFT-based peak detection may erroneously identify a frequency component with lower amplitude as the dominant peak due to the influence of spectral leakage. Since the heartbeat frequency is typically determined by locating the highest spectral peak, failing to correctly identify the true main frequency component can lead to substantial estimation errors.

To address this issue, we introduce a magnitude-first ordering strategy (hereafter referred to as the reordering strategy) applied after the final iteration of the FIIB algorithm. Upon completing the super-resolution estimation, all candidate frequency components are sorted in descending order based on their corresponding magnitudes. This ensures that the frequency component with the highest energy is selected as the final output for HR estimation.

Table 6 presents two representative scenarios. In Scenario 1, the unsorted algorithm mistakenly identified the interference peak at 1.37 Hz as the dominant frequency component, whereas the true HR was 1.20 Hz, an error that could lead to a misdiagnosis in a clinical setting. After applying the reordering strategy, the system correctly identified the HR peak at 1.21 Hz, with a deviation of only 0.01 Hz (approximately 0.6 bpm). Scenario 2 is even more notable: despite the presence of respiratory harmonic interference at 0.9 Hz, the reordering strategy successfully corrected HR from the erroneous output of 0.9 Hz to the fundamental HR frequency of 1.135 Hz, with an error controlled within 0.015 Hz (0.9 bpm). These results demonstrate that the reordering strategy significantly enhances the accuracy of frequency estimation.

### 4.3. Influence of the Measurement Environment

**Figure 13:**
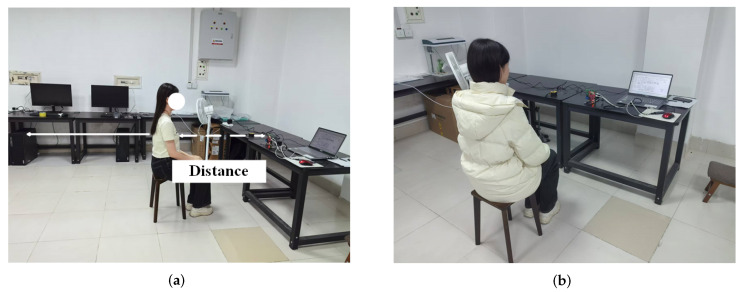
(**a**) Description of experimental scenes at different distances. (**b**) Experimental measurement scenario in heavy clothes.

In this section, we investigate the impact of different distances and varying clothing thicknesses on the HR estimation performance of the proposed system. Figure 13a illustrates the experimental setup, where volunteers are positioned at various distances from the radar.

Table 7 presents the RMSE values for six subjects at various distances. Our proposed method achieves RMSE values below 2.61 bpm in all cases. Notably, the proposed method achieves high accuracy within 1.5 m, with RMSE values consistently below 1.48 bpm. In particular, the average RMSE at 1 m is as low as 1.11 bpm, demonstrating the superior precision of the method. However, as the distance increases beyond 1.5 m, detection accuracy gradually deteriorates.

Next, we required the subject to wear thin clothing (approximately 120 g, as shown in Figure 13a) and thick clothing (approximately 1000 g, as shown in Figure 13b) to perform testing experiments at 1 m. Table 8 presents the RMSE results under different clothing thicknesses.

As shown in Table 8, clothing thickness has little impact on detection performance, with thin clothing yielding slightly better results, achieving an RMSE of 1.11 bpm compared to 1.38 bpm for thick clothing.

### 4.4. Overall Performance

**Figure 14:**
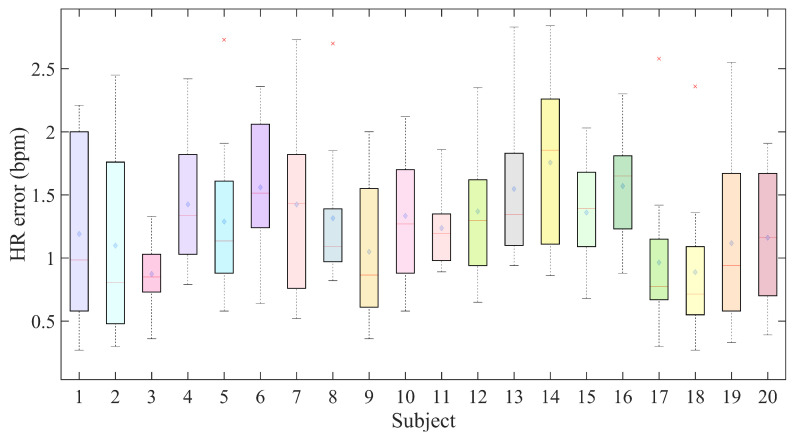
HR estimation biases for different users.

To assess the generalizability of the proposed heart rate detection system across a diverse population, we conducted experiments with 20 volunteers, including 10 males and 10 females. The participants’ body mass index (BMI) ranged from 18.5 to 27.3, encompassing categories from normal weight to slightly overweight, thereby ensuring a broad representation of body types. During all experiments, subjects were seated at a fixed distance of 1 m from the radar. Figure 14 displays box plots representing the distribution of HR estimation errors for each participant.

In Figure 14, blue diamonds indicate mean RMSE values, while red crosses represent outliers. The results show that, for most subjects, the HR estimation error lies between 0.5 and 2 bpm. The best performance achieved an average RMSE of 0.48 bpm, while the worst reached 2.65 bpm, resulting in an overall average RMSE of 1.28 bpm. These findings demonstrate the strong robustness of the proposed algorithm across individuals of varying gender and physical conditions, a critical factor for advancing FMCW radar applications in both clinical and home health monitoring settings.

### 4.5. Elderly Subjects and Three-Minute Data Testing

To assess the robustness of the proposed algorithm across different application scenarios, we performed extended experiments involving two distinct subject groups. The results are summarized in Table 9 and Table 10.

Table 9 details the 3-min group, consisting of 10 adult volunteers who each underwent a 3-min continuous recording. Table 10 presents the elderly group, comprising 10 volunteers with an average age of approximately 60 years, each recorded for 1 min. The two groups were independently recruited with no overlap of subjects. All experiments were conducted with a fixed radar-to-subject distance of 1 m.

As presented in Table 9 and Table 10, the RMSE values for the 3-min group range from 1.04 to 2.44 bpm, with a mean of 1.79 bpm. The error distribution is relatively concentrated, suggesting that the algorithm maintains robust stability during extended measurements. For the elderly group, RMSE values range from 0.55 to 1.94 bpm, with an average of 1.20 bpm. Several subjects exhibit particularly low errors, indicating that the algorithm adapts well to the elderly population. No significant outliers were observed in either group. Overall, the algorithm demonstrates strong robustness across varying recording durations and diverse subject groups.

### 4.6. Comparison with Other Literature

We further compared the performance of the proposed scheme with several recent studies, as summarized in Table 11. Reference [22] introduced an adaptive motion cancellation method based on FMCW virtual array radar, enabling real-time vital sign monitoring during substantial body movements. Their system achieved HR estimation errors within 3 bpm under static conditions. In [35], a likelihood estimation technique utilizing Newton’s method was proposed to enhance vital sign parameter accuracy, attaining an HR detection RMSE below 1.5 bpm. Reference [41] employed augmented differentiation and cross-multiplication to address phase discontinuities in cardiorespiratory signal extraction, followed by an improved variational pattern extraction method to retrieve respiratory and heartbeat waveforms. Sparse spectral representations were then applied to estimate RR and HR, resulting in an HR detection error of 3.4 bpm. Reference [42] developed a second-order-derivative-based algorithm for HR and RR monitoring, achieving an HR estimation error of 3.42 bpm with relatively low computational complexity.

In this study, our framework improves frequency estimation accuracy, achieving a mean RMSE below 2.61 bpm across various distances. Although reference [35] reported an RMSE under 1.5 bpm at 1.2 m, our approach attained an even lower RMSE below 1.48 bpm at the longer distance of 1.5 m, demonstrating superior detection accuracy.

Pan et al. proposed a hybrid method combining Frequency-Time Phase Regression (FTPR) and Time Windowed Variance (TWV) for heartbeat detection using Doppler radar [43]. Their approach achieved an RMSE around 0.9 bpm, showing enhanced frequency resolution over short windows. However, it is important to note that [43] only included six datasets collected from two subjects at a fixed radar distance of 0.5 m. In contrast, our study covers a wider range of experimental conditions, including diverse subject groups (e.g., elderly individuals), varied recording durations, and more realistic measurement scenarios.

## 5. Conclusions

In this paper, we propose a novel non-contact HR detection scheme based on MDCA and the FIIB algorithm. First, the proposed MDCA method significantly enhances the SNR by performing signal accumulation across both temporal and spatial domains. Extensive experimental results confirm the effectiveness of this approach. Second, we introduce FIIB for HR monitoring for the first time, achieving high-precision and super-resolution frequency estimation while maintaining low computational complexity. Experimental results show that FIIB improves HR detection accuracy by 1.08 bpm compared to the traditional FFT method.

To address misdetection issues of FIIB in vital sign signal analysis, we propose an effective magnitude-first reordering strategy that further improves the robustness of frequency estimation. Furthermore, through systematic analysis, we determine recommended values for key algorithm parameters—the number of effective frequency components L=4 and the number of iterations Q=10, providing practical guidance for real-world applications.

Comprehensive simulation and experimental evaluations demonstrate that the proposed scheme significantly outperforms conventional methods in HR estimation accuracy. Notably, the system achieves excellent precision, with a root mean squared error (RMSE) as low as 1.12 bpm at 1 m. Nevertheless, the method still exhibits certain limitations under motion conditions, which will be addressed in future work. Additionally, we plan to extend the approach to multi-target scenarios, with a particular focus on long-term health monitoring in complex home environments.

## Figures and Tables

**Table 1:** FIIB frequency estimation results.

Frequency Interval	Target	True (Hz)	Measurement (Hz)	RMSE (Hz)
0.75	$f_{1}$	1.05	1.0526	0.0026
	$f_{2}$	1.125	1.1231	0.0019
0.5	$f_{1}$	1.05	1.0507	0.0007
	$f_{2}$	1.10	1.0994	0.0006

**Table 2:** Radar parameters.

Parameter	Value
Start Frequency	77 GHz
Bandwidth	3.99 GHz
Frame Period	50 ms
Number of ADC Samples	200
Chirp Numbers	2
Idle Time	7 μs
Frame Frequency	20 Hz

**Table 3:** RMSE for different schemes. (RMSE unit: bpm).

Subject	Scheme 1	Scheme 2	Scheme 3	Scheme 4
1	2.01	4.24	3.25	2.73
2	4.33	2.12	2.30	1.74
3	3.52	3.15	3.03	2.88
4	2.41	1.98	1.82	1.49
5	3.01	3.64	3.24	2.72
6	2.06	1.93	2.11	1.75
7	4.91	2.48	2.30	2.18
8	2.30	2.79	2.33	2.24
9	3.33	2.94	2.70	2.40
10	3.36	2.67	2.48	2.24
11	5.30	5.42	3.86	2.98
12	4.67	4.58	3.53	2.43
13	23.18	15.85	3.42	2.36
14	2.55	2.18	2.27	2.09
15	4.00	6.45	3.14	2.82
16	2.09	1.86	1.80	1.89
17	2.24	2.27	2.12	1.74
18	2.35	2.42	2.02	1.65
19	2.30	2.06	2.06	2.03
20	6.15	3.27	2.20	1.67
**Average**	**4.30**	**3.72**	**2.60**	**2.20**

**Table 4:** RMSE of HR using different frequency measurements. (RMSE unit: bpm).

Subject	Scheme 4	Scheme 5	Scheme 6 (Proposed)
1	2.73	1.36	1.36
2	1.74	1.03	1.06
3	2.88	0.57	0.57
4	1.49	0.48	0.55
5	2.72	1.67	0.88
6	1.75	0.58	0.48
7	2.18	1.29	1.07
8	2.24	2.19	2.19
9	2.40	1.21	0.62
10	2.24	1.86	1.86
11	2.98	1.67	1.58
12	2.43	1.34	1.20
13	2.36	1.32	0.85
14	2.09	0.66	0.63
15	2.82	1.06	1.03
16	1.89	0.77	0.71
17	1.74	1.65	1.65
18	1.65	1.66	1.60
19	2.03	1.71	1.69
20	1.67	1.43	0.75
**Average**	**2.20**	**1.28**	**1.12**

**Table 5:** RMSE for HR measurements at different L parameters. (RMSE unit: bpm).

Group	L = 2	L = 4	L = 6	L = 8
1	1.62	1.46	1.87	1.91
2	0.87	0.74	0.83	0.89
3	0.93	0.70	0.94	1.36
4	0.88	0.88	0.89	0.70
5	0.82	0.74	1.14	1.67
6	1.12	1.00	0.85	0.85
7	2.58	2.36	2.55	2.45
8	1.35	1.21	1.73	1.73
9	0.91	0.91	0.97	0.67
10	0.85	0.85	1.03	1.12
**Average**	**1.19**	**1.09**	**1.28**	**1.33**

**Table 6:** HR detection results before and after reordering.

Unsorted (Hz)	Sorted (Hz)	Real HR (Hz)
Noise peak (1.37)	HR peak (1.21)	1.20
Respiratory harmonic (0.9)	Fundamental frequency of HR (1.135)	1.15

**Table 7:** RMSE at different distances. (RMSE unit: bpm).

Subject	0.5 m	1 m	1.5 m	2 m	2.5 m
1	1.06	0.85	1.21	2.00	1.06
2	1.45	1.12	2.09	2.54	3.67
3	0.85	1.36	1.51	2.98	4.42
4	1.48	1.48	1.89	1.67	2.48
5	1.26	1.09	1.05	1.89	2.70
6	1.30	0.74	1.13	1.84	1.30
**Average**	**1.23**	**1.11**	**1.48**	**2.15**	**2.61**

**Table 8:** RMSE under different clothing thicknesses. (RMSE unit: bpm).

Subject	Thin	Thick
1	0.85	1.21
2	1.12	1.12
3	1.36	1.68
4	1.48	1.76
5	1.09	1.52
6	0.74	0.96
**Average**	**1.11**	**1.38**

**Table 9:** RMSE Values of Heart Rate Estimation for the 3-Minute Group (Unit: bpm).

Subject	3-Minute
1	1.70
2	1.04
3	1.74
4	2.11
5	2.44
6	1.60
7	1.72
8	1.69
9	2.43
10	1.48
**Average**	**1.79**

**Table 10:** Heart Rate Estimation RMSE for Elderly Subjects (Unit: bpm).

Subject	Elderly
1	1.30
2	1.52
3	1.27
4	0.87
5	0.55
6	0.74
7	1.94
8	1.09
9	1.27
10	1.47
**Average**	**1.20**

**Table 11:** HR estimation results of our work and other references.

Reference	Radar	Range (m)	Test Time (s)	Error
[22]	77 GHz FMCW Radar	0.5–2.0	300	<3 bpm
[35]	77 GHz FMCW Radar	0.5–1.2	30	<1.5 bpm
[41]	77 GHz FMCW Radar	1–2	60	<3.4 bpm
[42]	60 GHz BGT60	0.5	30	3.42 bpm
[43]	77 GHz IWR1443Boost	0.5	120	0.9 bpm
Our work	77 GHz FMCW Radar	0.5–2.5	180	<2.61 bpm

## Data Availability

The datasets presented in this article are not readily available because they are part of an ongoing study. Requests to access the datasets should be directed to the corresponding author.
